# Pneumothorax catamenial: resultats de 18 cas opérés

**DOI:** 10.11604/pamj.2018.30.168.15308

**Published:** 2018-06-25

**Authors:** Raphael Ouede, Blaise Demine Alexandre, Ayegnon Kouakou Gregoire, Landry Kohou-kone, Edouard N’guessan, Maurice Gabin Kouacou, Jean-Marcel Ahui Brou, Flavien Kouassi Kendja, Yves Tanauh

**Affiliations:** 1 Service de Chirurgie Thoracique, Institut de Cardiologie d’Abidjan, Abidjan, Côte d’Ivoire; 2 Service d’Anesthésie et de Réanimation, Institut de Cardiologie d’Abidjan, Côte d’Ivoire; 3 Service de Gynéco-obstétrique, Centre Hospitalier Universitaire de Reichville, Côte d’Ivoire; 4 Service de Pneumo-Phtisiologie, Centre Hospitalier Universitaire de Cocody, Côte d’Ivoire

**Keywords:** Endométriose thoracique, pneumothorax cataménial, phrénoplastie, pleurodèse, hormonothérapie, chirurgie, Thoracic endometriosis, catamenial pneumothorax, phrenoplasty, pleurodesis, hormone therapy, surgery

## Abstract

L'objectif de notre étude était de proposer une approche thérapeutique du pneumothorax cataménial à partir des résultats de nos 18 cas opérés. Il s'agit d'une étude rétrospective de Janvier 1994 à Décembre 2016 qui a concerné 18 patientes âgées en moyenne de 32,2 ans opérées d'un pneumothorax cataménial droit (16 cas) et bilatéral (2 cas). Les patientes ont été réparties en 3 groupes en fonction de l'évolution dans le temps de notre attitude chirurgicale: le groupe 1 (G1) de janvier 1994 à juin 2006, le groupe 2 (G2) de juillet 2006 à février 2008 et le groupe 3(G3) de mars 2008 à décembre 2016, ces groupes contenaient respectivement 5, 2 et 11 patientes. Ces patientes toutes nullipares avaient une dysménorrhée depuis la puberté associée dans 11 cas à des douleurs thoraciques cataméniales. La radiographie standard du thorax a été systématique et complétée dans 8 cas par un scanner thoracique qui a objectivé en plus du pneumothorax, des bulles apicales (5 cas). L'exploration par mini thoracotomie postéro-latérale (16 cas) et par vidéothoracoscopie (2 cas de G3) a retrouvé des fenestrations diaphragmatiques (18 cas) et des bulles (5 cas). La biopsie des lésions et la résection des bulles a été systématique. Vis-à-vis des fenestrations diaphragmatiques, la chirurgie a consisté dans le groupe 1 en une résection-suture avec abrasion pleurale, dans le groupe 2 une couverture par un patch de Gore-tex avec abrasion pleurale et dans le groupe 3 une couverture par un patch avec talcage pleural. Une hormonothérapie (triptoreline) de 6 mois a été prescrite à chaque patiente en postopératoire pour suspendre les menstrues. Le résultat de la chirurgie a été apprécié sur la base de la survenue ou non d'une récidive du pneumothorax à la reprise des menstrues. La mortalité a été nulle. Le séjour hospitalier post opératoire moyen était de 9,32 jours. Les examens anatomopathologiques ont confirmé l'endométriose thoracique dans 9 cas. Après un suivi moyen de 5,3 ans, le résultat était bon chez 12 patientes (3/5 de G1, 1/2 de G2 et 8/11 de G3), 3 patientes de G3 ont continué de présenter des épisodes de dyspnée minime au début de quelques menstrues sans récidive radiologique, 3 patientes (2 de G1 et 1 de G2) ont récidivé et ont fait l'objet de reprise chirurgicale. En cas de pneumothorax cataménial avec fenestrations diaphragmatiques, nous proposons une phrénoplastie de recouvrement au patch associée à un talcage pleural et une hormonothérapie complémentaire concomitante de 6 mois.

## Abstract (en)

This study aims to propose a therapeutic approach for catamenial pneumothorax based on outcomes reported in 18 cases. We conducted a retrospective study of 18 female elderly patients with an average age of 32.2 years who had undergone surgery for right (16 cases) and bilateral catamenial pneumothorax (2 cases) from January 1994 to December 2016. The patients were divided into 3 groups on the basis of the evolution of our surgical capability over time: group 1(G1) from January 1994 to June 2006, group 2 (G2) from July 2006 to February 2008, group 3(G3) from March 2008 to December 2016, these groups were composed of 5, 2 and 11 patients respectively. All these patients were nulliparous who had suffered from dysmenorrhoea associated, in 11 cases, to catamenial chest pain since puberty. Standard radiographic evaluation of the chest was sistematically performed and complemented, in 8 cases, by chest CT scan that showed apical bubbles in addition to pneumothorax (5 cases). Exploration through posterolateral mini-thoracotomy (16 cases) and through videothoracoscopy (2 case of G3) showed diaphragmatic fenestrations (18 cases) and bubbles (5 cases). Biopsy of lesions as well as resection of the bubbles were sistematically performed . Surgical treatment of diaphragmatic fenestrations was based, in group 1, on resection-suture with pleural abrasion, in group 2, on Gore-tex patches coverage with pleural abrasion and, in group 3, on patch coverage with pleural talcage. Each patient underwent hormone therapy (triptoreline) for 6 months during postoperative period, in order to suspend menstruations. Surgical outcomes were evaluated on the basis of the recurrence or non-recurrence of a pneumothorax after resumption of menstruations. Mortality was zero. Postoperative hospital length of stay was 9.32 days. Anatomo-pathological examinations confirmed thoracic endometriosis in 9 cases. After a mean follow-up period of 5.3 years, outcomes were good in 12 patients (3/5 in G1, 1/2 in G2 and 8/11 in G3); 3 patients in G3 continued to have minimal episodes of dyspnoea at the beginning of some menstrual cycles without radiological evidence of recidivism, 3 patients (2 in G1 and 1 in G2) had recurrences requiring reoperation. We recommend phrenoplasty using patches associated with pleural talcage and complementary concomitant hormone therapy for 6 months in patients suffering from catamenial pneumothorax with diaphragmatic fenestrations.

## Introduction

Le pneumothorax cataménial est la première entité clinique de l'endométriose thoracique, c'est un pneumothorax spontané récidivant survenant entre la veille des menstrues et 48 à 72 heures après leur début [1]. Il survient chez la femme en âge de procréer. Il est rare et représente moins de 25% des pneumothorax spontanés chez ces femmes [1-3]. Le tissu endométrial ectopique en cause est généralement localisé au niveau de la plèvre viscérale et surtout diaphragmatique, un contexte d'endométriose pelvienne et d'infertilité est aussi très souvent retrouvé [4]. Son caractère récidivant aux menstrues et son association très fréquente à une infertilité font de lui une affection dramatique pour la femme africaine et pose un véritable problème thérapeutique. Sa prise en charge est multidisciplinaire impliquant pneumologues, gynécologues et chirurgiens thoraciques [5]. Le traitement chirurgical est justifié par les fréquentes récidives sous traitement médical seul [5]. L'attitude thérapeutique est variée et non encore consensuelle faute d'étude prospective de grande ampleur. Le but de notre étude est de rapporter notre expérience et proposer une approche thérapeutique à partir des résultats de nos 18 patientes opérées.

## Méthodes

Une étude rétrospective mono centrique de Janvier 1994 à Décembre 2015 a concerné 18 patientes opérées pour un pneumothorax cataménial. En fonction de l'évolution dans le temps de notre attitude chirurgicale, les patientes ont été réparties en 3 groupes: le groupe 1(G1) de Janvier 1994 à Juin 2006 comportait 5 patientes, le groupe 2 (G2) de Juillet 2006 à Février 2008 comportait 2 patientes et le groupe 3 (G3) de mars 2008 à décembre 2016 comportait 11 patientes. Ces patientes âgées en moyenne de 32,2 ans (avec des extrêmes de 19 et 45 ans) nous ont été adressées de la pneumologie (13 cas) et de la gynécologie (5 cas). Elles étaient toutes nullipares et présentaient des dysménorrhées primaires depuis la puberté. D'autres antécédents gynécologiques et thoraciques ont également été retrouvés (Tableau 1). A l'admission en chirurgie thoracique, les 5 patientes de la gynécologie étaient à leur premier épisode et ont subi initialement un drainage pleural associé dans un cas à une hystérectomie totale de sauvetage pour une récidive de myomes utérins très hémorragiques. Les 13 autres patientes étaient au moins à leur troisième récidive dont 11 homolatérales avec une dyspnée d'effort modérée et 2 bilatérales concomitantes dans un tableau d'angoisse et de détresse respiratoire qui ont nécessité un drainage pleural bilatéral en urgence. L'interrogatoire a retrouvé dans tous les cas une corrélation nette entre la survenue du pneumothorax et les menstrues. La radiographie standard systématique du thorax a objectivé 16 pneumothorax droit et 2 pneumothorax bilatéraux, un scanner thoracique réalisé dans 8 cas a confirmé le pneumothorax et a montré des bulles apicales droites dans 5 cas sans lésion parenchymateuse pulmonaire ni médiastinale associée.

**Tableau 1:** Les antécédents retrouvés chez les patientes opérées d’endométriose thoracique

	Nature des antecedents	Effectif	Pourcentage (%)
**Au niveau thoracique**	douleur thoracique cataméniale	11	61,11
	pseudo asthme cataménial	4	22,22
**Au niveau Gynécologique**	nullipare	18	100
	dysménorrhée depuis la puberté	18	100
	interruption volontaire de grossesse	2	11,11
	kystectomie ovarienne	2	11,11
	insufflation tubaire	2	11,11
	endométriose pelvienne	3	16,67
	myomectomie	1	05,56
**autres**	appendicectomie	1	05,56
	cure herniaire ombilicale	1	05,56

**Figure 1:**
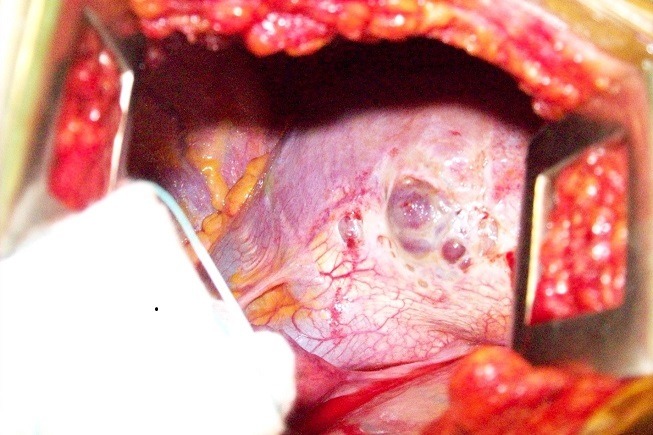
Une de nos images per opératoires montrant de multiples fenestrations diaphragmatiques chez une patiente présentant un pneumothorax cataménial

**Figure 2:**
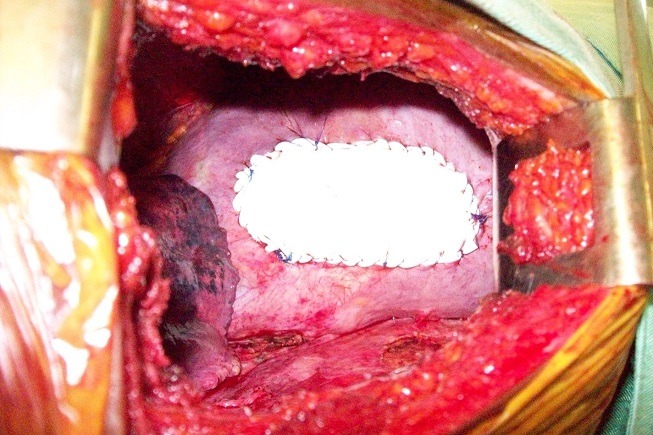
Une de nos images per opératoires montrant une phrénoplastie de recouvrement à la plaque de gore-tex chez une patiente présentant un pneumothorax cataménial

Une chirurgie a été réalisée chez ces patientes pendant la période péri menstruelle, sous anesthésie générale avec une intubation trachéo-bronchique sélective (13 cas) ou trachéale ordinaire (5 cas). La voie d'abord a été la thoracotomie postéro-latérale classique avec épargne musculaire mais 2 patientes du G3 ont fait l'objet d'une vidéothoracoscopie première qui a été convertie secondairement en thoracotomie à la découverte des fenestrations diaphragmatiques. Les côtés gauches ont été opérés 7 mois après le côté droit. L'exploration per opératoire a objectivé de multiples fenestrations de diamètre variable (infra centimétrique à 3 mm) au niveau du centre phrénique du diaphragme dans tous les cas sauf aux côtés gauches (Figure 1). Des bulles sur tout le poumon (5 cas), des zones brunâtres sur la plèvre pariétale (8 cas) et une pachypleurite viscérale engainant le poumon (2 cas) ont également été observées. La biopsie de toutes ces lésions suspectes a été systématique ainsi que la résection des bulles. Concernant les fenestrations diaphragmatiques, les gestes chirurgicaux ont consisté dans le G1 en une résection-suture avec symphyse pleurale par abrasion, dans le G2 une couverture par un patch en polyglactin de Gore-tex (phrénoplastie) avec abrasion pleurale (Figure 2) et dans le G3 une phrénoplastie avec symphyse pleurale par talcage. Deux drains pleuraux aspiratifs étaient mis en place en fin d'intervention. Un talcage pleural simple a été effectué aux côtés gauches. Les drains pleuraux sont restés en place jusqu'à une production liquidienne inférieure à 100 cc/24 heures, une absence de bullage pendant 48 heures confirmée par un test de clampage de 24 heures et une reexpansion pulmonaire satisfaisante à la radiographie thoracique de face. Les sorties ont été effectuées après une autre radiographie thoracique satisfaisante le lendemain de l'ablation des drains. Une hormonothérapie à la triptoreline de 6 mois a été prescrite par les gynécologues en post-opératoire à fin de suspendre les menstrues et permettre une consolidation de la symphyse pleurale; elle a été suivie correctement 13 fois, refusée 2 fois, abandonnée à 4 mois 3 fois (2 fois pour des raisons financières et une fois à cause des effets secondaires). Les patientes ont été suivies au moins jusqu'à 6 mois après la reprise complète des menstrues à l'arrêt du traitement hormonal. Le résultat de la chirurgie a été apprécié sur la base de la survenue ou non de récidive du pneumothorax à la reprise des menstrues.

## Résultats

La mortalité a été nulle. La morbidité postopératoire a été marquée par un retard de reexpansion pulmonaire résolutif par la kinésithérapie intense chez celle dont le poumon était engainé par une pachypleurite. Le séjour hospitalier moyen postopératoire a été de 9,32 jours avec des extrêmes de 6 et 19 jours. L'examen anatomopathologique a confirmé le diagnostic d'endométriose diaphragmatique dans 9 cas et pleurale viscérale dans 2 cas. Le suivi postopératoire a varié de 3 mois à 11 ans avec une moyenne de 5,3 ans. Douze patientes dont 3 des 5 de G1 1 des 2 de G2 et 8 des 11 de G3 ont eu une bonne évolution sans aucune gêne. Trois patientes de G3 ont continué de présenter des épisodes de dyspnée minime au début de quelques menstrues sans récidive radiologique. Trois patientes (16,7%) dont 2 des 5 de G1 et 1 des 2 de G2 ont récidivé: 1 controle latérale et 2 homolatérales (Tableau 2). Les récidives sont survenues 1 et 3 mois chez la deuxième patiente qui a refusé l'hormonothérapie et à 9 mois et 9 ans chez les autres. Trois reprises chirurgicales ont été effectuées: il a été réalisé respectivement à la première et deuxième récidive de celle qui en a eu 2, une suture itérative du diaphragme et un talcage pleural associés à une hormonothérapie après la deuxième récidive. Celle dont la récidive est survenue à 9 ans a subi une phrénoplastie de recouvrement avec talcage pleural, la dernière a été reprise à l'étranger. Dans tous les cas leur évolution a été bonne sans aucune gêne après les reprises.

**Tableau 2:** Tableau récapitulatif de l’évolution des pneumothorax cataméniaux opérés en fonction des gestes chirurgicaux et de l’observance de l’hormonothérapie

	N0	Age (ans)	Lésions observées	Gestes réalisés	Hormono-thérapie	évolution
Groupe 1	1	27	PD	R-S + Abrasion P.	Oui	bonne
	2	24	PD + blebs	R-S + Abrasion P.	refus	bonne
	3	25	PD	R-S + Abrasion P.	refus	2 récidives
	4	32	PD + blebs	R-S + Abrasion P.	oui	récidive
	5	19	Blebs	R-S blebs + Abrasion P.	oui	bonne
Groupe 2	6	31	PD	Plastie + Abrasion P.	oui	bonne
	7	37	PD	Plastie + Abrasion P.	4 mois	récidive
Groupe 3	8	25	PD	Plastie + talc	Oui	Bonne
	9	45	PD	Plastie + talc	4 mois	Dyspnée+/-
	10	36	PD	Plastie + talc	Oui	Bonne
	11	27	PD + blebs	Plastie + R-S blebs+talc	Oui	Bonne
	12	32	PD	Plastie + talc	Oui	Bonne
	13	37	PD + pachyP	Décor +Plastie + talc	Oui	Dyspnée +/-
	14	33	blebs	R-S blebs + talc	4 mois	Bonne
	15	38	PD	Plastie + talc	Oui	Dyspnée+/-
	16	30	PD	Plastie +talc	Oui	Bonne
	17	38	PD	Plastie + talc	Oui	Bonne
	18	44	PD	Plastie + talc	Oui	Bonne

PD = perforation diaphragmatique, pachyP = pachypleurite, R-S = resection-suture, abrasion P = abrasion pleurale pariétale, Talc = talcage pleural, décor = decortication pleurale viscérale, plastie = plastie diaphragmatique de recouvrement

## Discussion

Le pneumothorax cataménial est la plus fréquente (73% des cas) des 4 entités cliniques (pneumothorax, hémothorax, hémoptysie, nodule pulmonaire) de l'endométriose thoracique. L'endométriose thoracique désigne les manifestations cliniques respiratoires liées à la présence et aux modifications cycliques du tissu endométrial ectopique non néoplasique dans l'une des structures du thorax. Sa pathogénie n'est pas parfaitement élucidée [1-4]. L'atteinte prédomine à droite mais les formes gauches et bilatérales comme dans notre étude sont décrites [2-4]. L'âge moyen de survenu du pneumothorax cataménial avoisine 30 ans [1-4] comme dans notre étude (32,22 ans). Selon Jacques Klein et al. [6] cette affection est rare en Afrique et en Asie, mais son incidence semble sous-estimée vue sa méconnaissance par la plupart des praticiens. Nous avons opéré 18 cas en 21 ans, 11 de ces cas ont été étiquetés initialement comme une simple douleur thoracique accompagnant une dysménorrhée et 4 comme un pseudo asthme. Le diagnostic est souvent fait après plusieurs épisodes [6]. Cependant ce diagnostic est aisé lorsqu'on sait l'évoquer devant les manifestations respiratoires (douleur thoracique et de dyspnée de degré variable) pendant les règles [2, 3]. Le diagnostic étiologique par contre demeure un challenge car l'examen anatomopathologique n'est positif que dans 1/3 des cas [1, 2]. De ce fait, ce diagnostic étiologique n'est pas obligatoire pour le traitement du pneumothorax cataménial [4]. La pathogénèse du pneumothorax cataménial est encore discutée. L'hypothèse admise par la plupart des auteurs est la perforation pulmonaire et/ou diaphragmatique par les implants endométriaux. Trois mécanismes physiopathologiques combinables induits par l'effet des stéroïdes ovariens lui ont été attribués: il s'agit de la rupture spontanée des bulles, de la destruction alvéolaire périphérique et du passage trans-diaphragmatique d'air venu de l'extérieur via l'utérus, les trompes et la cavité péritonéale pendant les menstrues [2-4, 7, 8]. Le traitement hormonal basé surtout sur le Danazol et les analogues de la GnRH (gonadotropine releasing hormone) vise la réduction de la sécrétion des stéroïdes ovariens et la suspension des menstrues. Cette pseudo grossesse ou pseudo ménopause induite crée un environnement hormonal défavorable à la croissance et au maintien de l'endomètre ectopique et évite donc la survenue du pneumothorax cataménial [5, 9].

L'observance de cette hormonothérapie est difficile à cause parfois des effets secondaires mais surtout du désir de grossesse de ces patientes jeunes. En conséquence, le taux de récidive avec ce traitement médical seul avoisine 60% [2] d'où la nécessité d'un traitement chirurgical [5]. La chirurgie vise: d'abord la résection d'éventuelle bulle et de tous les tissus endométriaux visibles ce qui impose la pratique de ces interventions pendant la période des menstrues où le tissu endométrial est plus expressif [5], ensuite la réparation du poumon et/ou du diaphragme et enfin la symphyse pleurale. Les lésions les plus constantes et les plus inquiétantes sont les fenestrations diaphragmatiques [3]. Non traitées, en plus des récidives du pneumothorax qu'elles vont engendrer, ces fenestrations peuvent être à l'origine d'une hernie diaphragmatique [10, 11]. Plusieurs techniques de réparation de ce diaphragme perforé sont décrites, il peut être: agrafé, reséqué et suturé, collé à la colle biologique, plicaturé et ou recouvert par une prothèse [3, 5, 12, 13]. La symphyse pleurale a pour but de prévenir les récidives comme dans les autres pneumothorax spontanés. Cette symphyse pleurale est soit mécanique par abrasion de la plèvre pariétale ou par pleurectomie pariétale soit chimique par talcage [13-16]. L'association de plusieurs gestes chirurgicaux est recommandée pour réduire le risque de récidive. Plusieurs combinaisons sont discutées mais l'attitude optimale reste à définir [2, 3, 5, 12, 13, 15]. Nous, nous avons commencés par des résection-sutures du diaphragme associées à une abrasion pleurale pariétale (groupe 1). Nos résultats n'ont pas été satisfaisants (2 cas de récidive sur 5), mais ces résultats étaient superposables à ceux de la littérature où le taux de récidive avec ces techniques avoisinait 25% [5, 10, 12, 15]. Bagan [13], dont aucune des 5 patientes n'a récidivé après une phrénoplastie par recouvrement de la zone tendineuse du diaphragmatique avec une prothèse de polyglactin a proposé cette technique à fin de prendre en charge les fenestrations occultes. L'une des 2 patientes (groupe 2) chez qui nous avons réalisé cette technique associée à une abrasion pleurale a récidivé. Chez les 11 dernières patientes (groupe 3) nous avons associé à la phrénoplasties un talcage pleural et aucune récidive n'a été constatée. Concernant la voie d'abord chirurgicale, la vidéo-thoracoscopieest la technique de choix permettant de visualiser au mieux des anomalies pleuro-diaphragmatique (2). Mais la thoracotomie postéro-latérale avec épargne musculaire facilite les réparations du diaphragme quoi que délabrante [3, 14-16]. L'hormonothérapie décrite plus haut est recommandée actuellement en additif à la chirurgie pendant 6 à 9 mois [2, 3, 5, 8, 9, 13-16]. La suppression temporaire des menstrues qu'elle engendre donne le temps à la symphyse pleurale de bien se consolider. Elle doit débuter en postopératoire immédiat pour éviter d'éventuelles menstrues précoces qui font le lit de la récidive du pneumothorax [12]. Dans notre expérience, cette double prise en charge a été proposée systématiquement et expliquée à chacune de nos patientes.

## Conclusion

Le pneumothorax cataménial est une pathologie rare, l'intervention de la chirurgie dans sa prise en charge est récente dans nos pratiques. Notre attitude chirurgicale a subi des modifications sous l'influence d'une part, de nos propres résultats et d'autre part, des résultats et recommandations de la littérature. Cette chirurgie semble avoir trouvé ses pas au vue des résultats de notre courte série. Ainsi pour les pneumothorax cataméniaux avec des fenestrations diaphragmatiques, nous proposons: une intervention chirurgicale en période des menstrues, une résection d'éventuelle lésion endométriale pleuro-pulmonaire, une phrénoplastie de recouvrement au patch en polyglactin associée à un talcage pleural et une hormonothérapie additive concomitante de 6 mois.

### Etat des connaissances actuelle sur le sujet

- Les méthodes diagnostiques;
- La physiopathologie;
- Certaines méthodes thérapeutiques.

### Contribution de notre étude à la connaissance

- Une autre expérience mono centrique de plusieurs cas;
- D'autres protocoles thérapeutiques;
- L'évolution à moyen et long terme par protocole thérapeutique.

## Conflits d’intérêts

Les auteurs ne déclarent aucun conflit d'intérêt.
